# Interface Formation during the Growth of Phase Change Material Heterostructures Based on Ge-Rich Ge-Sb-Te Alloys

**DOI:** 10.3390/nano12061007

**Published:** 2022-03-18

**Authors:** Caroline Chèze, Flavia Righi Riva, Giulia Di Bella, Ernesto Placidi, Simone Prili, Marco Bertelli, Adriano Diaz Fattorini, Massimo Longo, Raffaella Calarco, Marco Bernasconi, Omar Abou El Kheir, Fabrizio Arciprete

**Affiliations:** 1 Dipartimento di Fisica, Università di Roma “Tor Vergata”, Via della Ricerca Scientifica 1, 00133 Rome, Italy; cheze_caroline@yahoo.fr (C.C.); giuliadibella05@gmail.com (G.D.B.); simone.prili@roma2.infn.it (S.P.); fabrizio.arciprete@roma2.infn.it (F.A.); 2 Department of Physics, Sapienza University of Rome, Piazzale Aldo Moro 5, 00185 Rome, Italy; ernesto.placidi@uniroma1.it; 3 Istituto per la Microelettronica e Microsistemi (IMM), Consiglio Nazionale delle Ricerche (CNR), Via del Fosso del Cavaliere 100, 00133 Rome, Italy; marco.bertelli@artov.imm.cnr.it (M.B.); adriano.diazfattorini@artov.imm.cnr.it (A.D.F.); massimo.longo@artov.imm.cnr.it (M.L.); raffaella.calarco@artov.imm.cnr.it (R.C.); 4 Department of Materials Science, University of Milano-Bicocca, Via R. Cozzi 55, 20125 Milan, Italy; marco.bernasconi@unimib.it (M.B.); o.abouelkheir@campus.unimib.it (O.A.E.K.)

**Keywords:** PCM, Ge-rich Ge-Sb-Te alloys, heterostructures, electronic properties

## Abstract

In this study, we present a full characterization of the electronic properties of phase change material (PCM) double-layered heterostructures deposited on silicon substrates. Thin films of amorphous Ge-rich Ge-Sb-Te (GGST) alloys were grown by physical vapor deposition on Sb_2_Te_3_ and on Ge_2_Sb_2_Te_5_ layers. The two heterostructures were characterized in situ by X-ray and ultraviolet photoemission spectroscopies (XPS and UPS) during the formation of the interface between the first and the second layer (top GGST film). The evolution of the composition across the heterostructure interface and information on interdiffusion were obtained. We found that, for both cases, the final composition of the GGST layer was close to Ge_2_SbTe_2_ (GST212), which is a thermodynamically favorable off-stoichiometry GeSbTe alloy in the Sb-GeTe pseudobinary of the ternary phase diagram. Density functional theory calculations allowed us to calculate the density of states for the valence band of the amorphous phase of GST212, which was in good agreement with the experimental valence bands measured in situ by UPS. The same heterostructures were characterized by X-ray diffraction as a function of the annealing temperature. Differences in the crystallization process are discussed on the basis of the photoemission results.

## 1. Introduction

Phase change material (PCM) devices based on chalcogenide alloys constitute a well-established technology for optical storage and non-volatile electronic memories [1,2]. Moreover, PCMs are currently attracting increasing interest as promising candidates for neuromorphic applications [3]. The reason for such growing attention can be found in the requirements of modern information technology, which relies on the implementation of a large network of electronic smart systems, also known as the Internet of Things (IoT). The IoT can connect people, places, and systems, with a potential huge impact on several aspects of everyday life [4]. The impressive amount of data and information generated by modern electronic systems still requires large numbers of fast, cheap, and power-efficient embedded non-volatile memories and processing devices. A particular field of IoT is that of automotive applications, which must comply with rather strict requirements for the embedded electronic devices since they are expected to work at a temperature of 165 °C for at least 10 years, without any data loss. PCM-based memories have been found to be a suitable technology for the realization of rewritable storage media also in the automotive field. The ternary (GeTe)_m_(Sb_2_Te_3_)_n_ alloys are widely used as active material for the fabrication of both optical and electrical memories, commonly in the composition Ge_2_Sb_2_Te_5_ (GST225) [5]. However, the GST225 crystallization temperature T_x_ of 150 °C is not sufficient to cope with the automotive requirements. Therefore, very recently, intense investigation into thermally stable PCMs with higher T_x_ has emerged [6,7]. GeSbTe with Ge-rich compositions (GGST) was found to be one of the most promising alloys [8]. However, thermally stable PCMs, such as GGST, suffer from a high write latency due to their low crystallization speed [9], and, in order to find a good compromise between stability and crystallization speed, new strategies have to be pursued. Investigations on superlattice structures made of alternating Sb_2_Te_3_ and GeTe or GST films [10,11,12,13] have revealed not only higher speed than GST, but also better thermal stability than Sb_2_Te_3_ films. This suggests that combining layers of materials with different physical properties can help to achieve the best material trade-off. Furthermore, investigations [10,13] have shown that the interfaces between the films in the superlattice, due to interdiffusion and alloying, can strongly affect the functional properties of the materials and consequently the overall performance of the final devices.

Here, following this strategy, we present a study on double-layer heterostructures based on GGST (high T_x_ but low crystallization speed) and Sb_2_Te_3_ or Ge_2_Sb_2_Te_5_ (higher crystallization speed). To obtain insights on the properties of the interface during its formation, the structures were grown by successive depositions of the two PCM films. The electronic properties of the samples were studied in situ by X-ray and ultraviolet photoemission spectroscopies (XPS and UPS), with a particular focus on the formation of the interface to reveal the presence of intermixing and/or interdiffusion phenomena possibly occurring between the two layers. Moreover, the amorphous (a-) to crystalline (x-) phase transition was investigated by means of ex situ X-ray diffraction (XRD) under annealing conditions at increasing temperatures, revealing different crystallization behaviors between the two heterostructures.

## 2. Experiment

### 2.1. Sample Growth

**Figure 1:**
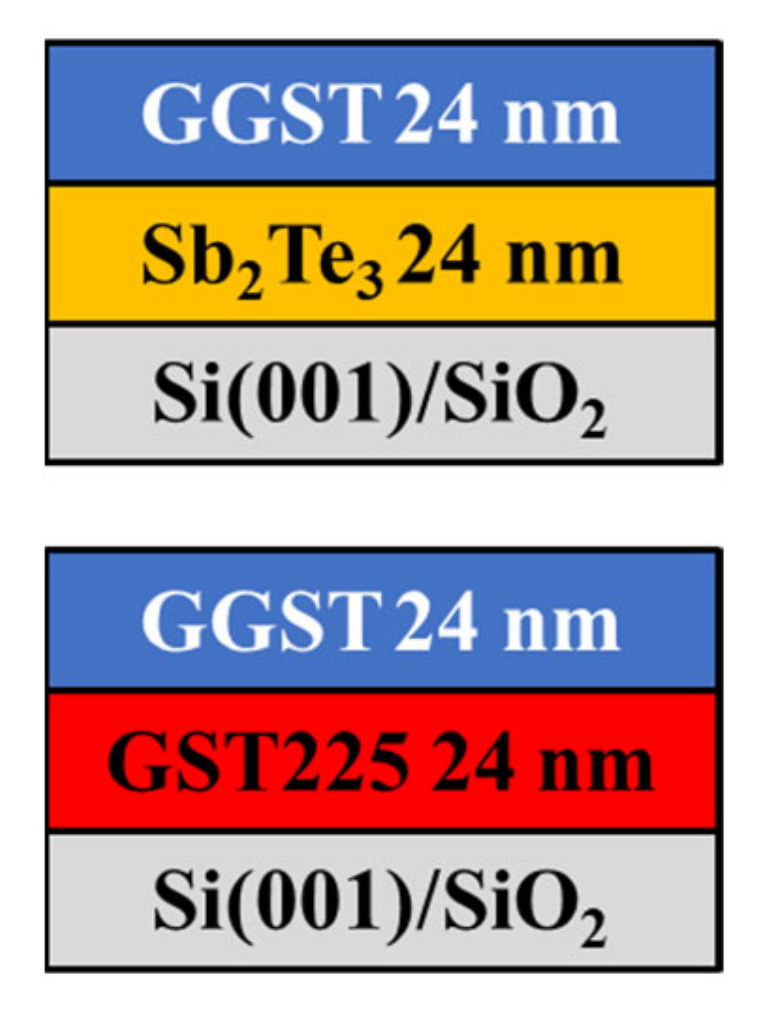
Schematics of the double-layer heterostructures.

The PCM films were deposited by Physical Vapor Deposition in a custom-made ultra-high vacuum (UHV) chamber system equipped with four Knudsen cells (Dr. Eberl MBE-Komponenten GmbH, Weil der Stadt, Germany) for the thermal evaporation of In (Azelis Electronics, Paris, France), Te, Sb and Ge (Alfa Aisar, Haverhill, MA, USA). The growth chamber was UHV-connected to the analysis chamber, allowing the in situ photoemission characterization of the samples. The two double-layered heterostructures investigated consisted of two PCM films of the same thickness deposited on Si(100)/SiO_2_ substrates. In order to study the formation of the interface during the growth of the heterostructure, after the deposition of the first PCM layers (24 nm of Sb_2_Te_3_ or GST225), we carried out successive partial depositions of GGST, for resulting thicknesses of 1, 2, 4, 8, 16, and 24 nm (sample A: Si(100)/SiO_2_/Sb_2_Te_3_(24 nm)/GGST(24 nm); sample B: Si(100)/SiO_2_/GST225(24 nm)/GGST(24 nm); see scheme in Figure 1), and characterized each partial deposition by XPS and UPS. The films were grown using stoichiometric flux ratios. The Sb_2_Te_3_ layer was grown at 200 °C—and therefore in its crystalline phase—while all the others were grown nominally in the a-phase by keeping the substrate at room temperature. During the growth, the substrate temperature was monitored by a thermocouple positioned close to the sample holder and the pressure was in the range of high 10^−9^ Torr. In the case of sample B, after the XPS/UPS characterization, and before the GGST layer depositions, the a-GST225 layer was annealed in UHV at 300 °C to promote crystallization.

### 2.2. Photoemission Characterization

For both sample A and B, photoemission experiments were performed in situ on the first PCM layer and after each partial deposition of the GGST layers.

XPS was carried out by using an Omicron DAR 400 Al/Mg Kα non-monochromatized X-ray (Taunusstein, Germany) source and a 100 mm hemispherical VG-CLAM2 electron spectrometer (Uckfield, UK). For all the XPS experiments, different core levels were considered: Te 3d, Sb 3d, Te 4d, Sb 4d, and Ge 3d acquired with the Mg Kα line, and Ge 2p_3/2_ acquired with the Al Kα line. Binding energies were calibrated by setting the Au 4f_7/2_ core level at 83.95 eV, measured on an Au foil in electrical contact with the sample. The collected XPS spectra were analyzed and fitted by the KolXPD software (version 1.8.0) and libraries (http://kolxpd.com accessed on 8 August 2018) using Voigt functions and Shirley background.

UPS measurements were carried out by using a VG helium discharge source set to excite the He I photon line.

### 2.3. X-ray Diffraction

XRD measurements were performed ex situ on replicas of sample A and B grown completely amorphous and after the deposition of a 10-nm-thick protective Si_3_N_4_ capping layer. XRD was carried out by a Bruker D8 Discover diffractometer (Billerica, MA, USA) equipped with a Cu X-ray source and an Anton Paar DHS1100 dome-type heating stage for temperature measurements in N_2_ atmosphere. For each sample, grazing incidence ω-2θ XRD scans were acquired at increasing annealing temperatures from 30 to 300 °C under nitrogen flow. Annealing of sample A was performed with 10 steps of duration of 57 min each at a heating rate of 50 °C/min. Annealing of sample B was performed with 12 steps of duration of 25 min each at a heating rate of 60 °C/min.

### 2.4. Density Functional Theory Simulations

As will be shown in the next sections, the growth of samples A and B led to the formation of a top GGST layer very close to the GST212 composition.

Therefore, we computed the electronic density of states for a 216-atom model of amorphous GST212 generated by quenching from melt within molecular dynamics simulations based on the Born–Oppenheimer approximation and the solution of the electronic problem within Density Functional Theory (DFT). We used the exchange and correlation functional due to Perdew, Burke, and Ernzerhof (PBE) [14] within the same framework employed in our previous works on GST225 [15] and GGST [16] alloys, as detailed in the Supplementary Materials.

The structural properties were computed by averaging over a 12 ps trajectory at constant temperature and constant volume. The structural properties of our model of amorphous GST212 are similar to those reported in a previous DFT work [17]. Partial pair correlation functions, bond angle distribution functions, and the distribution of coordination numbers at 300 K are reported in Figures S1–S3 in the Supplementary Materials. Average partial coordination numbers and the percentage of fraction of the different bonds are provided in Tables S1 and S2 in the Supplementary Materials, where we also report in Figure S4 the distribution of the q parameter, which allows the quantification of the fraction of Ge atoms in a tetrahedral bonding geometry. The distribution of rings is reported instead in Figure S5 in the Supplementary Materials. The structural parameters are overall in between those that we reported previously [16] for the two neighboring compositions, GST323 and GST423.

The electronic density of states (DOS) of the model optimized at zero temperature was computed from Kohn–Sham (KS) orbitals at the supercell Γ-point with the HSE06 hybrid functional [18] to better reproduce the band gap, as we have done in our previous works on GST225 [19,20]. KS energies were broadened by a Gaussian function with a variance of 80 meV.

## 3. Results and Discussion

**Figure 2:**
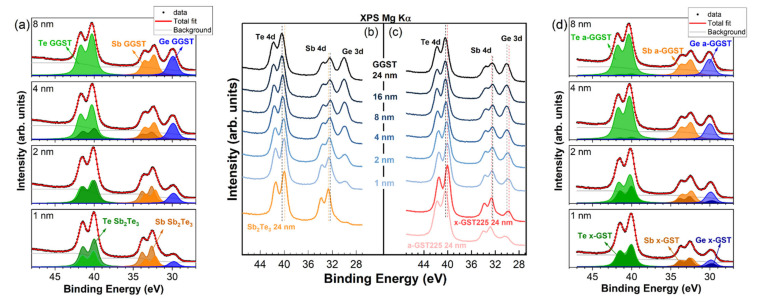
XPS spectra evolution of the Te 4d, Sb 4d, and Ge 3d core levels for (**b**) sample A, (**c**) sample B at increasing thicknesses of the GGST film. Chemical shifts toward higher and lower BEs are visible for Te 4d, Ge 3d, and Sb 4d, respectively. Fit deconvolution of the core levels for selected thicknesses (1, 2, 4, and 8 nm) of the deposited GGST layer is also shown for (**a**) sample A, (**d**) sample B.

A stack of Te 4d, Sb 4d, and Ge 3d XPS core levels as a function of the thickness of the deposited GGST layer is reported in Figure 2 for the grown heterostructures. Sb_2_Te_3_ was grown crystalline, while GST225 was grown amorphous and then annealed in UHV conditions at T = 300 °C before GGST deposition. Upon annealing (red curve in Figure 2c) all the core levels in the GST225 spectrum exhibited a chemical shift toward lower binding energy (BE) with respect to the as-grown a-GST225 (pink curve), being δ_Te 4d_ = −0.32 eV, δ_Sb 4d_ = −0.19 eV, and δ_Ge 3d_ = −0.75 eV. This behavior reflects the change in chemical bonding upon the transition from a- to x-phase, with a lowering of the covalent character during crystallization. In the following, we will continue to refer to this film as x-GST225 (see also below the discussion of valence band measurements). After deposition of GGST, chemical shifts of the Te 4d and Ge 3d core levels toward higher BEs were clearly visible in the XPS spectra of both the heterostructures, while the opposite trend could be observed for Sb 4d (Figure 2b,c). To understand the behavior of the chemical shifts after the deposition of the GGST layers, it is useful to consider the expected BE shifts after the formation of binary compounds [21,22,23]. The Sb-Te bonding shifts the Sb 4d core levels toward higher BE (+0.6 eV with respect to metallic Sb) and the Te 4d toward lower BE (−0.55 eV with respect to metallic Te). Conversely, the Ge-Te bonding moves the Te 4d core levels of around 0.3 eV towards lower BE and the Ge 3d core levels to higher BE with a shift of +0.55 eV [21,22,23]. Therefore, after the formation of a ternary Ge-Sb-Te alloy, the BE of Te 4d core levels should be in the middle between the values expected for metallic Te and Sb_2_Te_3_. The more the alloy is Ge-rich, the more Te 4d core levels are shifted towards higher BEs. The same holds for Ge 3d core levels. In the case of Sb 4d, shifts towards lower BEs are expected. The BE shifts observed in our samples agree with this general trend if we consider the expected absolute BE positions for pure Te 4d, Sb 4d, and Ge 3d core levels (40.6, 32.1, and 29.3 eV, respectively). Moreover, at increasing GGST coverages, we observe a progressive increase in the shifts. To interpret this behavior, we need to consider the surface sensitivity of XPS, which limits the signal coming from the first layer at increasing thicknesses of the second layer. As a matter of fact, the collected spectra are the superposition of a decaying contribution from the first layer and an increasing one from the second layer, giving rise to an apparent progressive shift. The decay of the contribution of the first layer will be slower in the case of the formation of a rough interface. However, a similar behavior would also suggest the formation, at the interface, of an alloy with increasing Ge content. To discriminate the formation of alloys of varying composition due to a possible intermixing at the interface of the heterostructures, a quantitative analysis of XPS spectra is needed. It is important to note that the Te 4d, Sb 4d, and Ge 3d core levels fall within a narrow BE interval between 25 and 50 eV (see Figure 2), meaning that the kinetic energies of the collected photoelectrons are almost the same, as well as their escape depth. At these energies, the escape depth of the electrons is of the order of 2–3 nm [24], and the quantitative analysis of XPS spectra is therefore representative of the bulk composition of the samples. The analysis of the Te 4d, Sb 4d, and Ge 3d core levels is thus particularly important because, by a fit deconvolution of the experimental spectra (normalized for the sensitivity factor of the specific element [24]), we can determine, in situ, the composition of the alloys as a function of the GGST thickness.

For the best fit deconvolution of the spectra, we modeled the system by means of two components for each of the elements in the alloys, one representing the contribution from the first PCM layer and the second one representing the GGST second layer. The first layer will be considered unchanged while the second layer will result as the average of the growing second layer and the intermixed interface, if any. Therefore, the best fits were obtained considering a vanishing contribution from the bottom PCM layers (Sb_2_Te_3_ or GST225) with fixed BEs, Lorentzian and Gaussian widths, spin–orbit splits, and branching ratios of the Voigt doublets.

In Figure 2a,d, the obtained results for selected representative thicknesses (1, 2, 4, 8 nm) of the deposited GGST film are reported for sample A and B, respectively. Within this model, if there is no intermixing at the interface, the components associated with the GGST growing layer would result at their final BE already after the first partial deposition (1 nm), regardless of the morphology of the interface. Therefore, the observation of a progressive shift at increasing GGST coverages suggests, in our case, a varying composition of the growing layer due to intermixing occurring at the interface during the evolution of the heterostructure. Obviously, intermixing involves both sides of the interface, but in our model, the intermixed layer is averaged in the contribution associated with the second layer.

**Figure 3:**
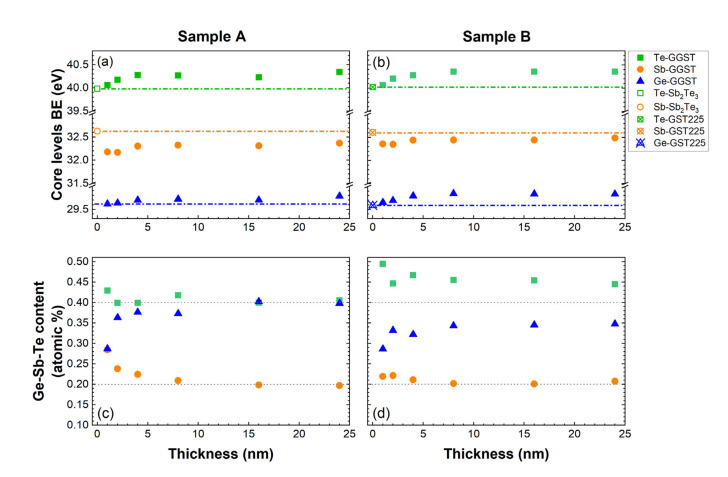
Core level BEs of the Sb_2_Te_3_ Te 4d and Sb 4d (open green square and orange circle, respectively), GST225 Te 4d, Sb 4d, Ge 3d (open crossed green square, orange circle, and blue triangle, respectively), and GGST Te 4d, Sb 4d, Ge 3d (green squares, orange circles, and blue triangles, respectively) for sample A (**a**) and sample B (**b**) as a function of the GGST layer thickness as estimated by fit deconvolution of the XPS spectra of Figure 1. The evolution of the composition of the GGST layer is shown for sample A (**c**) and sample B (**d**).

In Figure 3a,b, we plot the BE of the core levels for each element of the GGST growing layer for sample A and B, respectively, as determined from the fitting of the XPS spectra (light green, light orange, and blue curves in Figure 2a,d). The results clearly show that, for both heterostructures, the components associated with the second layer undergo a progressive shift in the first partial depositions, reaching a stable average value for GGST thicknesses higher than 4 nm. It is interesting to note that, while the final BE of the Ge and Te core levels are comparable for both heterostructures, some differences can be revealed in the behavior of the Sb core levels. After the deposition of 24 nm of GGST, the chemical shift toward lower BEs of Sb core levels is larger in sample A than in sample B. Moreover, after the very first depositions, for both heterostructures, the Sb 4d BE decreases toward the energy of metallic Sb and then increases again after 2 nm (a possible Sb segregation at the interface, not included in the model, cannot be ruled out). Again, this effect appears to be larger for sample A (see Figure 3a). These differences can be understood if we consider that, in heterostructure A, the first layer does not contain Ge, revealing the high sensitivity of the Sb 4d core levels to the Ge content.

To obtain further insights on this behavior and on the interface formation, the stoichiometry as a function of the thickness of the second layer was determined from the fitting of the core levels. The results are summarized in Figure 3c,d, where the compositions in atomic percentage are reported. For sample A, starting from the Sb_2_Te_3_ layer, such atomic percentages are compatible with composition Sb:2.0, Te:2.6, and the GGST layer continuously enriches in Ge and Te, from approximately Ge:2.0, Sb:2.0, Te:3.0 up to the final composition Ge:4.0, Sb:2.0, Te:4.1. In the case of sample B, the GST225 layer with composition Ge:1.1, Sb:2.0, Te:6.3 in the a-phase becomes a crystalline mixed 124/225 (Ge:1.3, Sb:2.0, Te:4.6) after annealing; as a matter of fact, the 124 composition is the most stable among the alloys along the pseudo-binary line [25,26]. The composition of the GGST layer evolves to a final Ge:3.4, Sb:2.0, Te:4.5, a value rather close to 424 as for the case of Sb_2_Te_3_/GGST. Considering these results, the slightly larger BE variation observed for Sb core levels after the deposition of the 24-nm-thick GGST layer in sample A can be explained with the formation of a slightly Ge-richer final composition. For both the heterostructures, the composition of the GGST layer progressively changes (see Tables S5 and S6 in the Supplementary Materials), reaching a stable average value for thicknesses higher than approximately 8 and 4 nm for sample A and B, respectively. The observed evolution of composition is compatible with the occurrence of intermixing phenomena at the interface between the two PCM layers, a hypothesis also supported by the progressive chemical shifts of the GGST core levels. By comparing the results in Figure 3c,d and considering the probing depth of XPS at these energies (which is approximately 2–3 nm), we can suggest that the intermixed region is larger in sample A than in B.

**Figure 4:**
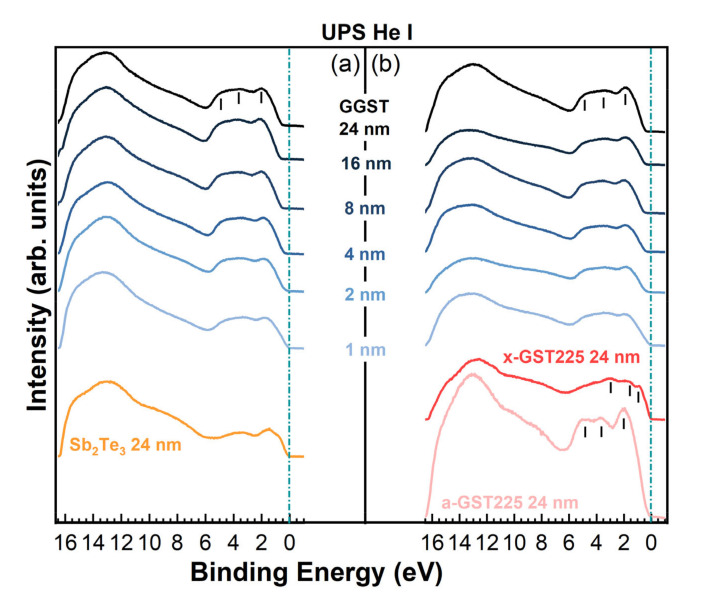
UPS spectra evolution measured for (**a**) sample A, (**b**) sample B as a function of the thickness of the deposited layers.

As for the XPS experiments, UPS spectra of the heterostructures were collected during the formation of the interface after each deposition step of the GGST layer. The results are summarized in Figure 4, where a change in the UPS spectra line-shape is visible for both heterostructures already after the deposition of 1 nm of GGST, with the valence band maximum (VBM) progressively shifting towards higher BEs at increasing thicknesses (and increasing Ge content) of the GGST overlayer from 1 to 24 nm. The VB of the 24-nm-thick GGST has a mixed Te 5p, Sb 5p, and Ge 4p character in the region between 6 and 0 eV and, for both sample A and B, is characterized by a prominent peak at approximately 2 eV, and two broad features at approximately 3.5 and 5 eV. The same line-shape can be observed in the UPS spectrum of the as-grown a-GST225 layer of sample B (pink curve in Figure 3), with the VB showing the same three main features around 2.0, 3.5, and 5.0 eV, typical features of amorphous GST [27]. Upon annealing (red curve in Figure 4b), GST225 crystallizes and the VB shows a change in the line-shape with the formation of clear peaks at approximately 0.7, 1.8, and 3.0 eV of BE and the energy of the VBM shifting toward the Fermi level indicated by the green line in Figure 4. Such a shift can be ascribed to an increased hole concentration due to the formation of vacancies in the Ge/Sb sublattice [27,28,29]. Interestingly, the experimental spectra for annealed x-GST225 have very close correspondence with the DOS calculated by DFT for an ordered trigonal t-GST structure [29]. The a- to x-transition of GST225 is also visible in the XPS spectra in Figure 2c (red curves). These observations, associated with a mixed 124/225 composition, suggest that annealing in UHV at 300 °C is effective in triggering the transition of a-GST225 to the trigonal phase. Similar results for the VB have been found by Klein et al. [28] for cubic GST124, Caravati et. al. for cubic GST225 [19,29], and by Akola and Jones [30] for cubic Ge-rich GST8 2 11.

A good correspondence with simulations was found also in the case of sample A for the Sb_2_Te_3_ VB (orange curve in Figure 4), which compares well to the theoretical DOS of crystalline Sb_2_Te_3_ [31,32] and to the experimental VB measured in situ for a polycrystalline sample [33], showing a mixed Te-Sb p character in the region 0–6 eV and a dominant s character in the range 7–13 eV.

**Figure 5:**
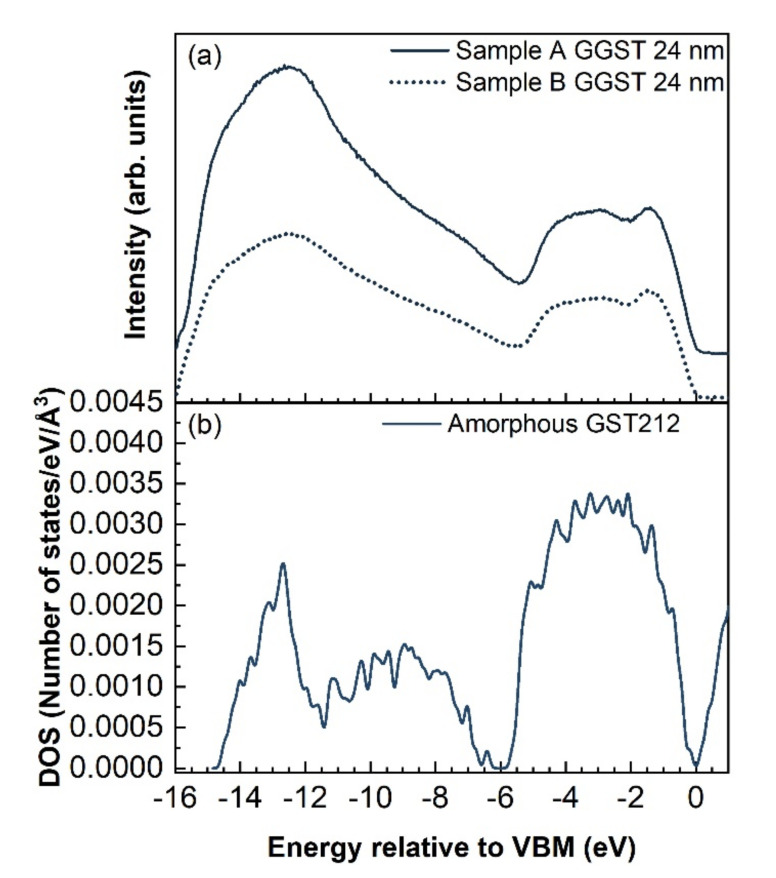
Comparison between (**a**) UPS spectra acquired on the two heterostructures after deposition of 24 nm of GGST and (**b**) DFT calculations of the DOS of amorphous GST212.

By inspection of the UPS spectra collected on the 24-nm-thick GGST films of both heterostructures, a shift of approximately 0.11 eV of the VBM towards higher BEs could be detected for sample A with respect to B, which is reasonable if we consider that sample B has a slightly lower Ge content, as determined by XPS [34]. It is also worth noting that the deposition of only 1 nm of GGST changes the VB line-shape of both samples, which shows the typical features of amorphous GST. The VBM shifts progressively as a function of GGST thickness up 8 nm and 2/4 nm for samples A and B, respectively, suggesting that the interface of sample B is sharper than the interface of sample A. Since the final composition of the GGST films obtained by XPS is close to 424 for both heterostructures, the VB measured by UPS for the 24 nm GGST layers was compared to the electronic DOS calculated by DFT simulations for amorphous GST212, as shown in Figure 5b. Experimental and theoretical spectra show quite good correspondence; we remark that previous DFT calculations [35] have revealed that alloys on the Sb-GeTe isoelectronic line (on average three p-electrons per atom) are particularly stable in the cubic crystalline phase. Their free energy is in fact very close to the convex hull built from the thermodynamically stable compositions [35]. This feature has resulted from the construction of a map of the formation free energy of GST alloys in the central part of the ternary phase diagram generated by high-throughput DFT calculations in Ref. [35]. We might expect that compositions on the Sb-GeTe line, such as GST212, are also more stable than others in the ternary phase diagram in the amorphous phase as well, although we do not have a map similar to that of Ref. [35] for the amorphous phase. Should this be the case, it would justify the formation of the GST212 composition after the growth of samples A and B reported above.

**Figure 6:**
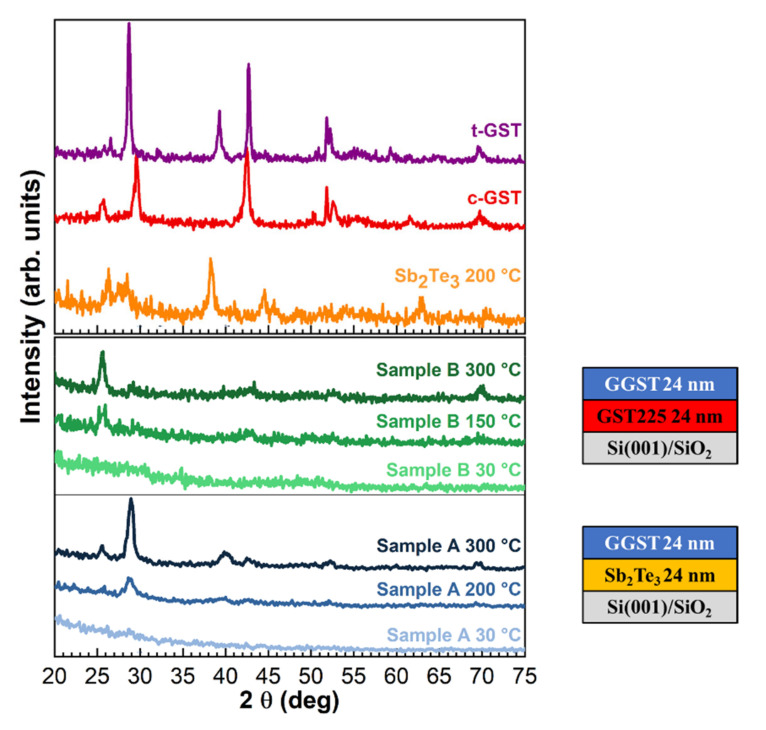
ω-2θ XRD curves as a function of the annealing temperature for sample A and B. Single layer (Sb_2_Te_3_, c-GST225, and t-GST225 used as reference for cubic and trigonal polycrystalline GST) XRD scans are shown as references.

The a- to x-transition of the two heterostructures was investigated by ex situ XRD measurements performed at increasing temperatures on replicas of samples A and B grown completely amorphous. The diffractograms as a function of annealing temperature are reported in Figure 6 for sample A at the selected temperatures of 30, 200, and 300 °C (blue curves) and for sample B at 30, 150, and 300 °C (green curves). The XRD curves collected on single layers of Sb_2_Te_3_ (orange curve), cubic c-GST225 (red curve), and trigonal t-GST225 (violet curve) as reference diffractograms for cubic and trigonal polycrystalline GST are also shown. As shown in Figure 6, the diffractograms of the heterostructures do not display any peak at 30 °C, thus confirming that the samples were both grown amorphous. A first visible difference between the two samples is the crystallization temperature, which is lower for sample B (T_x_ ~ 150 °C) than for sample A (T_x_ ~ 200 °C), coherent with the fact that the GGST layer in this latter sample is richer in Ge. In the case of sample B annealed at 150 °C, four diffraction peaks can be seen at 2θ = 25.7°, 29.6°, 42.7°, 69.7° that can be, respectively, identified as (111), (002), (022), (024) Bragg reflections of the c-GST. Interestingly, the contribution of the (111) and (024) reflections remains stable up to T = 350 °C (data not shown), while the other orientations disappear. In the case of sample A, the peak around 40°, identified as the (106) reflection of t-GST, is found together with the reflections at 2θ = 25.7°, 29°, 42.5°, 69.6° of c-GST at 200 °C. Upon a further increase in the temperature up to 300 °C, the contributions of the c-GST(002) and t-GST(106) reflections increase in intensity, while the c-GST(022) reflection remains almost stable. Furthermore, a faint reflection from c-GST(222) can be identified at 52.2°. These observations suggest that the crystallization of the two heterostructures occurs with different behaviors: while a mixed cubic–trigonal phase at around 200 °C is found for sample A, sample B shows a transition to the cubic phase already at around 150 °C. The second layer of sample A is Ge-richer than sample B and this can justify a higher T_x_, although a Tx > 250 °C would be expected for GGST [8]. This behavior is remarkable and suggests that the crystallization of the GGST can be affected by the presence of the underlayer. In particular, the introduction of a Sb_2_Te_3_ (sample A) or GST225 (sample B) underlayer beneath the GGST layer induces a lowering of the crystallization temperature in comparison with GGST single layers. Furthermore, although there are several reports that phase separation can occur upon annealing of GGST single layers, leading to the crystallization of Ge and other GST phases [9,36,37], no evidence of Ge segregation could be found in the XRD curves collected on our GGST-based heterostructures at increasing annealing temperature. All these observations suggest that the nature of the first layer and the extent of the interface can affect the crystallization behavior upon annealing. From the collected data, sample B results to be more stable at increasing annealing temperatures, which could be related to the fact that this system is characterized by a sharper interface than Sb_2_Te_3_/GGST, as suggested by the XPS and UPS results.

## 4. Conclusions

In this work, we present a full photoemission characterization of the interface formation in two double-layer PCM heterostructures: Sb_2_Te_3_/GGST and GST225/GGST.

The evolution of the heterostructure was followed as a function of the thickness of the GGST film. Interdiffusion between the GGST layer and the underlayer was highlighted for both cases, and to a larger extent for Sb_2_Te_3_/GGST. At increasing thicknesses of the GGST layer, a progressive increase in the Ge and Te content was found, which led to a final composition close to GST212 for both heterostructures. Once again, from the XPS measurements, evidence of the crystallization of the first GST225 layer could be found for sample B upon annealing in UHV, leading to a mixed GST124/GST225 composition and to a clear change in the VB line-shape. UPS measurements performed on the a-GGST layers also revealed quite good agreement between the experimental spectra and the DOS of amorphous GST212 calculated by DFT.

From the XRD data recorded upon annealing of both heterostructures, different behavior during the crystallization of the two systems could be found, with sample B resulting in a more stable heterostructure at increasing annealing temperatures. Furthermore, XRD measurements suggested that Sb_2_Te_3_ can act as a template for the crystallization of the GGST layer, leading for sample A to the formation of a mixed cubic–trigonal phase, unlike sample B, which maintained a stable cubic phase. Considering these results, it will be of interest to study multiple PCM layers and investigate the effect of the layer thickness on the crystallization process [38,39].

## Data Availability

The data that support the findings of this study are available from the corresponding author upon reasonable request.

## Supplementary Materials

The following supporting information can be downloaded at: https://www.mdpi.com/article/10.3390/nano12061007/s1, Figure S1: Total and partial pair correlation functions of the DFT model of amorphous GST212 at 300 K. The dashed lines indicate the bonding cutoff used to define the coordination numbers, which is 3.2 Å for all pairs except for Sb-Te for which the bonding cutoff was set to 3.4 Å; Figure S2: Bond angle distribution function of the DFT model of amorphous GST212 at 300 K. The total distribution and the distributions resolved for the different types of the central atom are shown; Figure S3: Distribution of the coordination numbers of the DFT model of amorphous GST212 obtained by using the bonding cutoff defined by the partial pair correlation functions in Figure S1; Figure S4: Distribution of the local order parameter q for tetrahedricity resolved for atomic species and coordination number for amorphous GST212. To obtain a continuous distribution, the order parameter for individual atoms is broadened with a Gaussian function 0.005 wide. The local order parameter q was introduced in [8] and it is defined by $q\;=\;1-\frac{3}{8}\;\sum_{i<k}\left[\frac{1}{3}+{\cos\theta }_{\mathrm{ijk}\;}\right]$ where the sum runs over the couples of atoms bonded to a central atom j and forming a bonding angle θ_ijk_. The order parameter evaluates q = 1 for the ideal tetrahedral geometry and q = 5/8 for a four-coordinated defective octahedral site. As shown in our previous work [9], the integration of the q distribution of the 4-coordinated Ge atoms from 0.8 to 1.0 gives a measure of the fraction of Ge atoms tetrahedrally coordinated, which turns out to be 45.2% for GST212; Figure S5: Ring distribution function of amorphous GST212; Table S1: Average coordination numbers for different pairs of atoms computed from the partial pair corre-lation functions (Figure S1) for the DFT model of amorphous GST212; Table S2: Percentage fraction of the different types of bonds in the DFT model of amorphous GST212; Table S3: Binding energies, amplitudes, Lorentzian widths, Gaussian widths of the Te 4d, Sb 4d and Ge 3d core levels as a function of the deposited thickness for sample A; Table S4: Binding energies, amplitudes, Lorentzian widths, Gaussian widths of the Te 4d, Sb 4d and Ge 3d core levels as a function of the deposited thickness for sample B; Table S5: Evolution of the composition across heterostructure A; Table S6: Evolution of the composition across heterostructure B [14,16,40,41,42,43,44,45,46].
