# Supplementary material for: Interface Formation during the Growth of Phase Change Material Heterostructures Based on Ge-Rich Ge-Sb-Te Alloys
Source: Nanomaterials (Basel). 2022 Mar 18;12(6):1007. doi: 10.3390/nano12061007 (PMC8949867; doi:10.3390/nano12061007)
Supplement: Supplementary file 1 [file nanomaterials-12-01007-s001.zip › nanomaterials-1610269-supplementary.pdf]

Supplementary Materials

Interface Formation during the Growth of Phase Change Material Heterostructures Based on Ge-Rich Ge-Sb-Te Alloys

Caroline Chèze [1], Flavia Righi Riva [1,*], Giulia Di Bella [1,2], Ernesto Placidi [2], Simone Prili [1], Marco Bertelli [3], Adriano Diaz Fattorini [3], Massimo Longo [3], Raffaella Calarco [3], Marco Bernasconi [4], Omar Abou El Kheir [4] and Fabrizio Arciprete [1]

[1] Dipartimento di Fisica, Università di Roma "Tor Vergata", Via della Ricerca Scientifica 1, 00133 Rome, Italy; cheze_caroline@yahoo.fr (C.C.); giuliadibella05@gmail.com (G.D.B.); simone.prili@roma2.infn.it (S.P.); fabrizio.arciprete@roma2.infn.it (F.A.)

[2] Department of Physics, Sapienza University of Rome, Piazzale Aldo Moro 5, 00185 Rome, Italy; ernesto.placidi@uniroma1.it

[3] Istituto per la Microelettronica e Microsistemi (IMM), Consiglio Nazionale delle Ricerche (CNR), Via del Fosso del Cavaliere 100, 00133 Rome, Italy; marco.bertelli@artov.imm.cnr.it (M.B.); adriano.diazfattorini@artov.imm.cnr.it (A.D.F.); massimo.longo@artov.imm.cnr.it (M.L.); raffaella.calarco@artov.imm.cnr.it (R.C.)

[4] Department of Materials Science, University of Milano-Bicocca, Via R. Cozzi 55, 20125 Milan, Italy; marco.bernasconi@unimib.it (M.B.); o.abouelkheir@campus.unimib.it (O.A.E.K.)

* Correspondence: flavia.righiriva@roma2.infn.it

1. DFT Calculations

DFT calculations were performed by using the exchange and correlation functional due to Perdew, Burke, an Ernzerhof (PBE) [1] and the norm conserving pseudopotentials from Goedeker, Teter, and Hutter [2,3]. Kohn-Sham (KS) orbitals were expanded in Gaussian-type orbitals of a valence triple-zeta-valence plus polarization basis set, while a basis set of plane waves up to a kinetic energy cutoff of 100 Ry was used to represent the charge density as implemented in the CP2k package (http://www.cp2k.org) [4]. We also included the semiempirical correction due to Grimme [5] for van der Waals interactions, as we have done in our recent work on other GGST alloys [6]. A model of liquid GST212 in a cubic 216-atom supercell was first equilibrated at 2000 K for 20 ps. A time step of 2 fs was used in the molecular dynamics simulations. The initial configuration was chosen as a special quasirandom structure of the metastable cubic phase of GST alloys with the cationic (anionic) sublattice occupied by 86 atoms of Ge (Te) and 22 atoms of Sb resulting in an actual composition of $Ge_2Sb_{1.02}Te_2$. The initial density was 0.0318 atoms/Å^3, which is 8% lower than the density of the cubic phase as it was reported for GST225 [7]. Then, we performed a second equilibration at 1000 K for 18 ps. The amorphous model was then generated by quenching the liquid from 1000 to 300 K in 100 ps at constant pressure with only isotropic changes of the simulation cell. The resulting equilibrium density at 300 K was 0.0313 atoms/Å^3, obtained as the average density of a 6 ps trajectory at constant pressure. The structural properties were computed by averaging over a 12 ps trajectory at constant temperature and constant volume. Finally, the atomic geometry was optimized at zero temperature at fixed volume to compute the electronic density of states reported in the article.

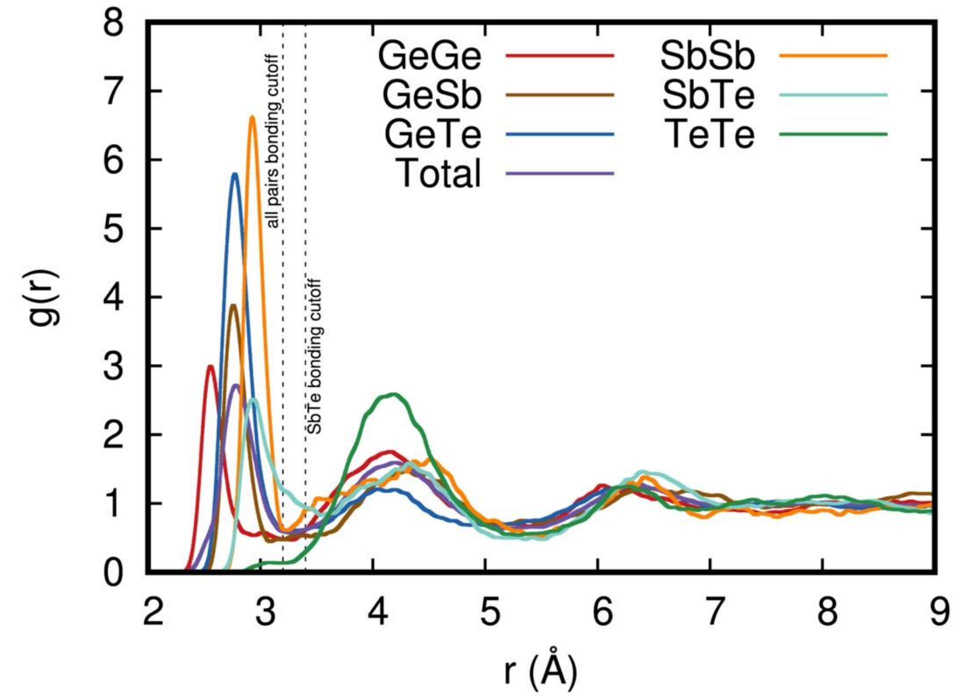

Figure S1. Total and partial pair correlation functions of the DFT model of amorphous GST212 at 300 K. The dashed lines indicate the bonding cutoff used to define the coordination numbers, which is 3.2 Å for all pairs except for Sb-Te for which the bonding cutoff was set to 3.4 Å.

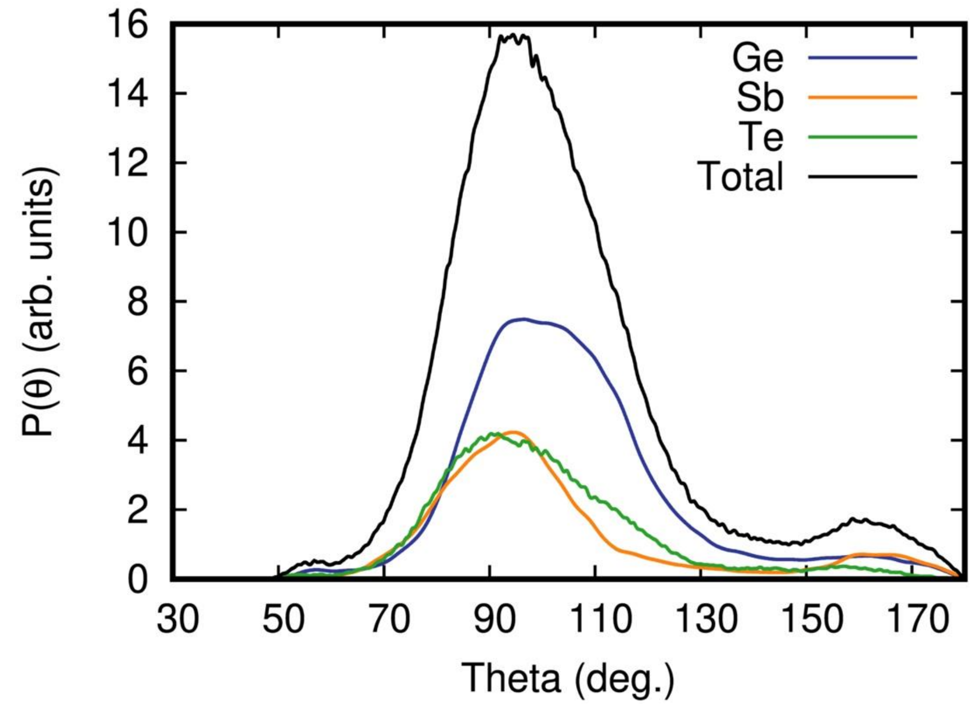

Figure S2. Bond angle distribution function of the DFT model of amorphous GST212 at 300 K. The total distribution and the distributions resolved for the different types of the central atom are shown.

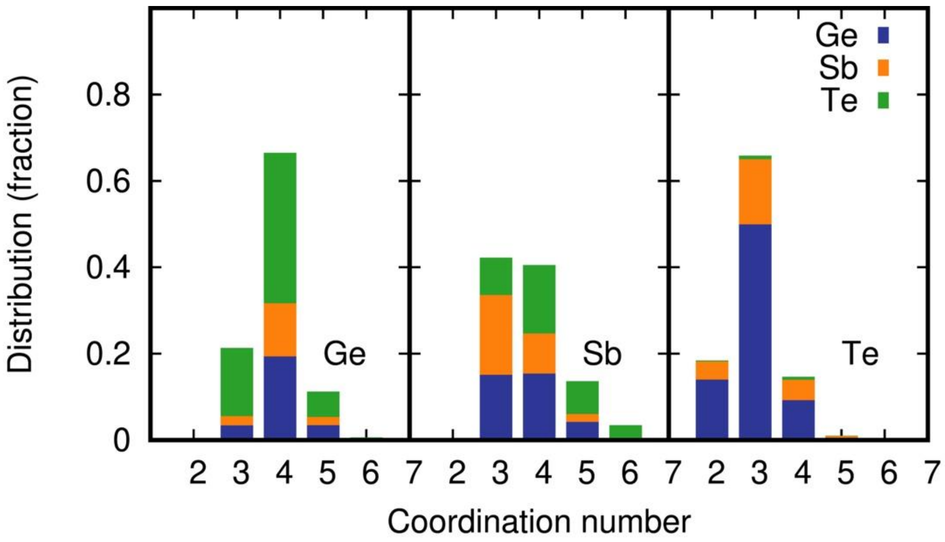

Figure S3. Distribution of the coordination numbers of the DFT model of amorphous GST212 obtained by using the bonding cutoff defined by the partial pair correlation functions in Figure S1.

Table S1. Average coordination numbers for different pairs of atoms computed from the partial pair correlation functions (Figure S1) for the DFT model of amorphous GST212.

-	Total	With Ge	With Sb	With Te
Ge	3.90	1.06	0.66	2.18
Sb	3.78	1.29	1.03	1.46
Te	2.98	2.18	0.75	0.05

Table S2. Percentage fraction of the different types of bonds in the DFT model of amorphous GST212.

-	Ge	Sb	Te
Ge	12.06	14.99	49.45
Sb	-	5.97	16.90
Te	-	-	0.63

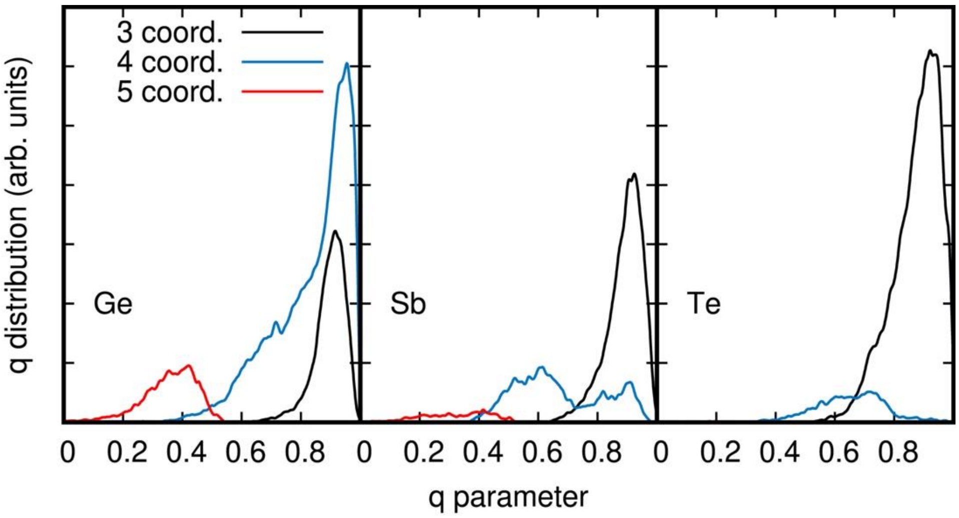

Figure S4. Distribution of the local order parameter q for tetrahedricity resolved for atomic species and coordination number for amorphous GST212. To obtain a continuous distribution, the order

parameter for individual atoms is broadened with a Gaussian function 0.005 wide. The local order parameter q was introduced in [8] and it is defined by $q = 1 - \frac{3}{8}\sum_{i<k}\left[\frac{1}{3} + \cos\theta_{ijk}\right]$ where the sum runs over the couples of atoms bonded to a central atom j and forming a bonding angle θ_{ijk}. The order parameter evaluates q = 1 for the ideal tetrahedral geometry and q = 5/8 for a four-coordinated defective octahedral site. As shown in our previous work [9], the integration of the q distribution of the 4-coordinated Ge atoms from 0.8 to 1.0 gives a measure of the fraction of Ge atoms tetrahedrally coordinated, which turns out to be 45.2% for GST212.

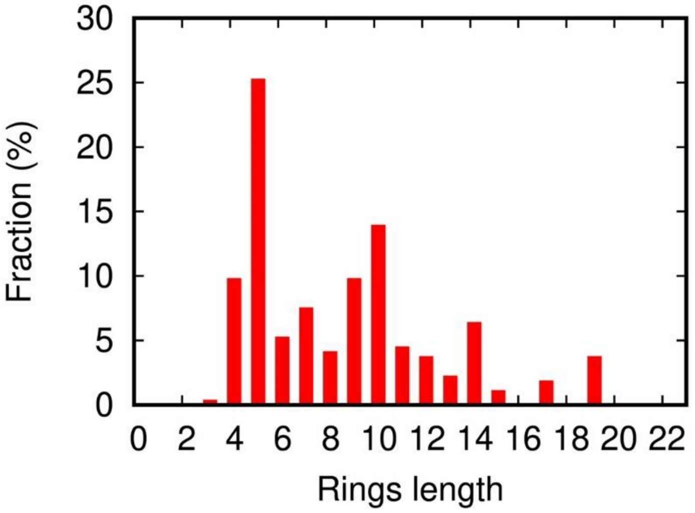

Figure S5. Ring distribution function of amorphous GST212.

2. XPS Fit Results

Table S3. Binding energies, amplitudes, Lorentzian widths, Gaussian widths of the Te 4d, Sb 4d and Ge 3d core levels as a function of the deposited thickness for sample A.

Phase	Core level	BE (eV)	Amplitude	L-width	G-width
Sb_2Te_3	Te $4d_{5/2}$	39.98	3104.3	0.62	0.69
	Sb $4d_{5/2}$	32.63	1938.2	0.62	0.59
+1 nm GGST	Te $4d_{5/2}$	40.06	1064.48	0.62	0.74
	Sb $4d_{5/2}$	32.18	582.2	0.62	0.47
	Ge $3d_{5/2}$	29.62	242.3	0.55	0.70
+2 nm GGST	Te $4d_{5/2}$	40.17	1274.2	0.62	0.79
	Sb $4d_{5/2}$	32.17	626.8	0.62	0.53
	Ge $3d_{5/2}$	29.64	394.2	0.55	0.80
+4 nm GGST	Te $4d_{5/2}$	40.28	1832.5	0.62	0.78
	Sb $4d_{5/2}$	32.30	850.5	0.62	0.74
	Ge $3d_{5/2}$	29.70	587.8	0.55	0.75
+8 nm GGST	Te $4d_{5/2}$	40.27	2252.8	0.62	0.79
	Sb $4d_{5/2}$	32.32	932.3	0.62	0.78
	Ge $3d_{5/2}$	29.72	683.9	0.55	0.78
+16 nm GGST	Te $4d_{5/2}$	40.23	2296.2	0.62	0.86
	Sb $4d_{5/2}$	32.37	941.0	0.62	0.84
	Ge $3d_{5/2}$	29.70	786.8	0.55	0.85
+24 nm GGST	Te $4d_{5/2}$	40.34	2340.9	0.62	0.86
	Sb $4d_{5/2}$	32.38	940.6	0.62	0.83
	Ge $3d_{5/2}$	29.78	783.2	0.55	0.86

Table S4. Binding energies, amplitudes, Lorentzian widths, Gaussian widths of the Te 4d, Sb 4d and Ge 3d core levels as a function of the deposited thickness for sample B.

Phase	Core level	BE (eV)	Amplitude	L-width	G-width
a-GST225	Te $4d_{5/2}$	40.34	3359.2	0.65	0.92
	Sb $4d_{5/2}$	32.81	788.2	0.66	0.65
	Ge $3d_{5/2}$	30.34	195.1	0.46	0.74
x-GST225	Te $4d_{5/2}$	40.02	3167.0	0.65	0.70
	Sb $4d_{5/2}$	32.61	1131.1	0.66	0.54
	Ge $3d_{5/2}$	29.59	291.6	0.46	0.66
+1 nm GGST	Te $4d_{5/2}$	40.07	1458.4	0.65	0.83
	Sb $4d_{5/2}$	32.35	353.5	0.66	0.75
	Ge $3d_{5/2}$	29.65	287.6	0.46	0.85
+2 nm GGST	Te $4d_{5/2}$	40.20	1742.3	0.65	0.80
	Sb $4d_{5/2}$	32.35	713.1	0.66	0.75
	Ge $3d_{5/2}$	29.70	440.3	0.46	0.85
+4 nm GGST	Te $4d_{5/2}$	40.27	2492.2	0.65	0.79
	Sb $4d_{5/2}$	32.44	932.8	0.66	0.75
	Ge $3d_{5/2}$	29.79	585.0	0.46	0.85
+8 nm GGST	Te $4d_{5/2}$	40.35	2614.2	0.65	0.80
	Sb $4d_{5/2}$	32.44	956.9	0.66	0.76
	Ge $3d_{5/2}$	29.85	670.9	0.46	0.85
+16 nm GGST	Te $4d_{5/2}$	40.35	2479.6	0.65	0.86
	Sb $4d_{5/2}$	32.44	907.7	0.66	0.76
	Ge $3d_{5/2}$	29.84	642.0	0.46	0.85
+24 nm GGST	Te $4d_{5/2}$	40.35	2597.5	0.65	0.82
	Sb $4d_{5/2}$	32.49	1001.9	0.66	0.86
	Ge $3d_{5/2}$	29.84	690.5	0.46	0.90

Table S5. Evolution of the composition across heterostructure A.

Phase	%Ge	%Sb	%Te
Sb_2Te_3	--	0.43	0.57
+1 nm GGST	0.29	0.28	0.43
+2 nm GGST	0.36	0.24	0.40
+4 nm GGST	0.38	0.22	0.40
+8 nm GGST	0.37	0.21	0.42
+16 nm GGST	0.40	0.20	0.40
+24 nm GGST	0.40	0.20	0.40

Table S6. Evolution of the composition across heterostructure B.

Phase	%Ge	%Sb	%Te
a-GST225	0.12	0.21	0.67
x-GST225	0.17	0.25	0.58
+1 nm GGST	0.29	0.22	0.49
+2 nm GGST	0.33	0.22	0.45
+4 nm GGST	0.32	0.21	0.47
+8 nm GGST	0.34	0.20	0.46
+16 nm GGST	0.35	0.20	0.45

+24 nm GGST	0.35	0.21	0.44

References

1. Perdew, J.P.; Burke, K.; Ernzerhof, M. Generalized gradient approximation made simple. *Phys. Rev. Lett.* **1996**, *77*, 3865. https://doi.org/10.1103/PhysRevLett.77.3865.
2. Goedecker, S.; Teter, M.; Hutter, J. Separable dual-space Gaussian pseudopotentials. *Phys. Rev. B* **1996**, *54*, 1703–1710, https://doi.org/10.1103/physrevb.54.1703.
3. Krack, M. Pseudopotentials for H to Kr optimized for gradient-corrected exchange-correlation functionals. *Theor. Chim. Acta* **2005**, *114*, 145–152, https://doi.org/10.1007/s00214-005-0655-y.
4. VandeVondele, J.; Krack, M.; Mohamed, F.; Parrinello, M.; Chassaing, T.; Hutter, J. Quickstep: Fast and accurate density functional calculations using a mixed Gaussian and plane waves approach. *Comput. Phys. Commun.* **2005**, *167*, 103–128, https://doi.org/10.1016/j.cpc.2004.12.014.
5. Grimme, S.; Antony, J.; Ehrlich, S.; Krieg, H. A consistent and accurate ab initio parametrization of density functional dispersion correction (DFT-D) for the 94 elements H-Pu. *J. Chem. Phys.* **2010**, *132*, 154104–154119. https://doi.org/10.1063/1.3382344.
6. Abou El Kheir, O.; Dragoni, D.; Bernasconi, M. Density functional simulations of decomposition pathways of Ge-rich GeSbTe alloys for phase change memories. *Phys. Rev. Mater.* **2021**, *5*, 095004, https://doi.org/10.1103/physrevmaterials.5.095004.
7. Njoroge, W.K.; Wöltgens, H.-W.; Wuttig, M. Density changes upon crystallization of Ge2Sb2.04Te4.74 films. *J. Vac. Sci. Technol. A: Vacuum, Surfaces, Films* **2002**, *20*, 230–233, https://doi.org/10.1116/1.1430249.
8. Errington, J.R.; DeBenedetti, P.G. Relationship between structural order and the anomalies of liquid water. *Nature* **2001**, *409*, 318–321, https://doi.org/10.1038/35053024.
9. Sosso, G.C.; Caravati, S.; Mazzarello, R.; Bernasconi, M. Raman spectra of cubic and amorphous Ge2Sb2Te5from first principles. *Phys. Rev. B* **2011**, *83*, https://doi.org/10.1103/physrevb.83.134201.
